# Residual Prolapse in Patients with III-IV Degree Haemorrhoids Undergoing Stapled Haemorrhoidopexy with CPH34 HV: Results of an Italian Multicentric Clinical Study

**DOI:** 10.1155/2014/710128

**Published:** 2014-06-15

**Authors:** Giuliano Reboa, Marco Gipponi, Andrea Rattaro, Giovanni Ciotta, Marco Tarantello, Angelo Caviglia, Antonio Pagliazzo, Luigi Masoni, Giuseppe Caldarelli, Fabio Gaj, Bruno Masci, Andrea Verdi

**Affiliations:** ^1^Coloproctology Unit, Casa di Cura San Camillo, Forte dei Marmi, Lucca, Italy; ^2^General Surgery and Breast Unit, IRCCS “San Martino-IST”, L.go R. Benzi 10, 16132 Genoa, Italy; ^3^Casa di Cura Triolo-Zancla, Palermo, Italy; ^4^Colo-Proctology Unit, San Camillo Hospital, Rome, Italy; ^5^General Surgery, Villa Paideia Hospital, Rome, Italy; ^6^General Surgery, Celio Military Hospital, Rome, Italy; ^7^General Surgery, Policlinico Umberto I, Rome, Italy; ^8^General Surgery, San Carlo IDI Hospital, Rome, Italy; ^9^General Surgery, Fatebenefratelli Hospital, Rome, Italy

## Abstract

CPH34 HV, a high volume stapler, was tested in order to assess its safety and efficacy in reducing residual/recurrent haemorrhoids. The clinical charts of 430 patients with third- to fourth-degree haemorrhoids undergoing SH in 2012-2013 were consecutively reviewed, excluding those with obstructed defecation (rectocele >2 cm; Wexner's score >15). Follow-up was scheduled at six and 12 months. Rectal prolapse exceeding more than half of CAD was reported in 341 patients (79.3%); one technical failure was reported (0.2%) without any serious untoward effect; and 1.3 stitch/patient (SD, 1.7) was required to achieve complete haemostasis. Doughnuts volume was higher (13.8 mL; SD, 1.5) in patients with a large rectal prolapse than with smaller one (8.9 mL; SD, 0.7) (*P* value <0.05). Residual and recurrent haemorrhoids occurred in 8 of 430 patients (1.8%) and 5 of 254 patients (1.9%), respectively. A high index of patient satisfaction (visual analogue scale = 8.9; SD, 0.9) coupled with a persistent reduction of constipation scores (CSS = 5.0, SD, 2.2) was observed. The wider prolapse resection well correlated with a clear-cut reduction of haemorrhoidal relapse, a high index of patient satisfaction, and clinically relevant reduction of constipations scores coupled with satisfactory haemostatic properties of CPH34 HV.

## 1. Introduction

Haemorrhoids represent one of the most frequent proctologic diseases, ranging in the adult population from 4% to 34% [1]. Bleeding during or soon after evacuation, anal pain and/or discomfort, and haemorrhoidal prolapse are the most common findings. According to the “*Unitary Theory of Rectal Prolapse,*” haemorrhoids are determined by an internal rectal prolapse that can be limited to the rectal mucosa (*mucosal prolapse*) or involve the muscle wall (*full-thickness rectal prolapse*) as well [2]. During defecation, this internal prolapse can descend down to the anal canal, up to or even beyond the anal verge, thus pushing-out anorectal mucosa and haemorrhoids. This dynamic prolapse weakens over time the supporting structures, such as* Treitz's* and* Parks'* ligaments, with a progressive sliding down of the haemorrhoids which is primarily due to the internal rectoanal prolapse.

Stapled haemorrhoidopexy (SH), by correcting the inherent internal rectal prolapse, achieves not only less postoperative pain, superior functional recovery with earlier return to normal activities, and improved patient satisfaction with respect to conventional haemorrhoidectomy (CH), but it can also ameliorate the symptoms of obstructed defecation, frequently reported in these patients, thus representing a standard of treatment [3–11]. However, currently available stapler devices pose some questions as to the extent of rectal prolapsed that can be actually resected, especially in patients with a large internal rectal prolapse, that is, a prolapse exceeding more than half of the length of the circular anal dilator (CAD). In such instances, a less than optimal prolapse resection increases up to 29.4% the rate of haemorrhoidal relapse after SH [12]. In order to accomplish a more satisfactory prolapse resection, stapled transanal rectal resection (STARR) was proposed as a surgical option to overcome such technological limitations, with a significant reduction of residual and/or recurrent haemorrhoids [12, 13].

Recently, a new device for transanal stapler-assisted surgery, CVPH34 HV, has been developed in order to guarantee a wider prolapse resection as compared to most of currently available staplers thanks to the high volume (HV) of the stapler casing (25 cm^3^). Its safety as well as the higher volume of resection was experimentally confirmed, with a significant increase in both volume and weight of the tissue specimens as compared to PPH03-33 (*P* = 0.0402 and *P* = 0.0375, resp.), with the latter having approximately 35% less volume of resection [14].

On these grounds, a retrospective observational multicenter clinical study was undertaken in patients with haemorrhoidal prolapse undergoing SH by means of CPH34 HV with the aim of assessing its safety and efficacy, with special care to the haemostatic properties of this HV stapler as well as the adequacy of prolapse resection.

## 2. Patients and Methods

The clinical charts of 430 patients with symptomatic third- or fourth-degree haemorrhoids, 18 to 80 years of age, who underwent SH in the period 2012-2013, were consecutively reviewed. The following Surgical Centers participated in the study: (i) Coloproctology Unit, Casa di Cura San Camillo, Forte dei Marmi, Lucca (*n* = 228); (ii) General Surgery, Casa di Cura Triolo-Zancla, Palermo (*n* = 74); (iii) Colon-Proctology Unit, San Camillo Hospital, Rome (*n* = 25); (iv) General Surgery, Villa Paideia Hospital, Rome (*n* = 25); (v) General Surgery, Celio Military Hospital, Rome (*n* = 9); (vi) General Surgery, Policlinico Umberto I, Rome (*n* = 30); (vii) Surgical Division, San Carlo Nancy Hospital, Rome (*n* = 13); and (viii) Surgical Division, Fatebenefratelli, Rome, Italy (*n* = 26).

The study group consisted of 209 (48,6%) males and 221 (51.4%) females with a mean age of 51 years (SD, 13.4 years; range, 19–80 years). All patients underwent complete preoperative proctologic examination, with flexible colonoscopy performed according to age, risk factors for colorectal cancer, and associated bowel symptoms. The clinical characteristics of patients are reported in Table 1. Patients with symptoms of obstructed defecation syndrome (ODS) who had an internal rectal prolapse associated with second-degree rectocele (2–4 cm) and a Wexner's constipation score more than 15 did not undergo SH but were eligible for the STARR procedure [15].

Patients usually underwent a one-day surgical procedure, with a preoperative self-administered rectal enema on the evening before and the morning of the operation; no antibiotic prophylaxis was given. Each patient gave his/her written informed consent and the study protocol was submitted to the Ethic Committee approval.

Preoperative clinical data included (i) specific symptoms of haemorrhoids such as pain (Visual Analogue Scale, VAS = 0–10); (ii) bleeding; (iii) haemorrhoidal prolapse/swelling; (iv) Wexner's Constipation Scoring System (CSS = 0–30); and (v) Goligher's classification of haemorrhoids (III or IV degree). Perioperative data included (i) operative time; (ii) surgical team; (iii) intraoperative assessment of the extent of internal rectal prolapse; (iv) associated procedures, such as excision of skin tags, excision of anal fissure, fistulotomy/fistulectomy, etc.); (v) technical failures of the stapler; (vi) specimen sizes (length, height, and volume); (vii) early complications (within 7 days), such as spontaneous or postdefecation anal pain, bleeding, urinary retention, faecal impaction, faecal urgency, and haemorrhoidal thrombosis; (viii) inpatient stay (days), and (ix) early reoperations (within 30 days) due to bleeding, haemorrhoidal thrombosis, dehiscence of the staple line, severe anal pain, or abscess.

Clinical follow-up consisted of outpatient visits that were scheduled at six and 12 months after the operation, as soon as the complete healing was achieved. Residual prolapse was defined as the reduction, without disappearance, of prolapsed tissue (haemorrhoids and/or rectal prolapse) within six months after the operation; recurrent disease was defined as the reappearance of prolapsed tissue after a symptom-free period of at least six months. Moreover, clinical follow-up data included (i) postoperative complaints such as urgency, itching, mucous discharge haemorrhoidal thrombosis, and faecal incontinence (grade I: gas; grade II: liquid stool, grade III: solid stool); (ii) late postoperative complications such as local or systemic infections, anal fissures, anal fistula, rectovaginal fistula, residual skin tags, and anorectal stenosis; (iii) associated symptoms of recurrent haemorrhoidal disease, such as anal pain (spontaneous and/or postdefecation: VAS = 0–10) or bleeding; (iv) grade of satisfaction (VAS = 0–10), and (v) the CSS score (range: 0–30).

### 2.1. Surgical Details

The operation was always performed by surgeons who were well trained in stapler-assisted transanal surgery, having performed at least 50 SH and 30 STARR procedures. Patients usually underwent spinal anaesthesia and were placed in a lithotomic position with a Trendelenburg*'s* tilt. Controlled digital stretching was performed initially with two fingers (index fingers) introduced carefully inside the anus and performing moderate traction laterally (gradually separating the two index fingers) and in an anteroposterior direction with fingers stretched (taking care not to hook the muscles of the pelvic floor). Then, the fingers were moved in a circular motion around the anus to gently break the inner sphincter fibres. Afterwards, two fingers on each hand were inserted repeating the circular motion to increase anal dilatation. Then, the lubricated circular anal dilator (CAD) was inserted with an obturator, an integral part of the CPH34 HV kit (Frankenman International Limited, Hong Kong). This was sutured to the perianal skin with four stitches. Once the obturator was removed, an intraoperative assessment of the rectal prolapse was accomplished in order to define whether it involved more than half of the length of the CAD. This parameter is clinically relevant because when the internal rectal prolapse exceeds this limit it means that the volume of the prolapse is higher than 14 cm^3^ and this is highly predictive of a less than optimal prolapse resection with a standard PPH device, whose volume of the casing is 17.4 cm^3^ [13]. A surgical anoscope was then inserted into the lumen of the CAD and a 2–0 Prolene purse-string suture was undertaken about 4 to 5 cm above the dentate line, to make the suture line at the end of the procedure approximately 2 to 3 cm proximal to the dentate line. The head of the circular stapler was introduced fully open proximal to the purse-string, which was tied with a closing knot; the ends of the suture were then pulled through the lateral holes of the instrument.

When the “Parachute” technique was used instead of a traditional Longo's procedure (i.e., with the single purse-string suture), six separated stitches at 3, 9, 1, 11, 5, and 7 hours or 12, 6, 2, 5, 7, and 10 hours were placed proximally at the same distance from the dentate line, as previously described. The single suture threads were secured to each other in two groups in order to allow them to be retrieved through the lateral suture conduits positioned on the right and left side of the circular stapler [16].

With both procedures, the ends of the sutures were fixed externally using a clamp and a gentle digital pressure on the sutures was maintained while tightening the stapler to draw the prolapsed rectal wall into the stapler casing. Hence, the stapler was fired in order to perform the prolapsectomy and rectopexy, having completed all necessary check to avoid rectovaginal fistula. Once the stapler was removed, the integrity of the mucosal cylinder removed (doughnut) was checked measuring into the operative room the specimen measures (length, mm; height, mm, and volume, mL with a graduated ampulla half filled with water) and then sent for histological examination. Haemostatic stitches were placed along the suture line in resorbable material (Vicryl 3–0) when required, and their number was recorded into the operative description. After prolonged observation to check for haemostasis, an absorbable plug was placed into the anal canal, thus concluding the intervention.

## 3. Results

Intraoperatively, 341 (79.3%) patients out of 430 had an internal rectal prolapse exceeding more than half of the length of the CAD while 89 (20.7%) had a rectal prolapse within half of the length of the CAD. A standard Longo's procedure was performed in the great majority of patients (*n* = 394; 91.6%) while the “Parachute” technique was used in 36 patients (8.4%). The latter technique was almost exclusively adopted at the Colon-Proctology Unit, San Camillo Hospital, Rome. The mean operative time was 26.1 (SD, 6.9; range, 15–60) minutes. One technical failure of the device did occur (0.2%) without any untoward effect as for the operation; only in a minority of patients haemostatic stitches were required to achieve complete haemostasis of the suture line, with a mean number of 1.3 stitch/patient (SD, 1.7; range, 0–7). Associated procedures were performed in 168 (39%) of patients, such as skin tags excision (*n* = 73; 43.4%), anal fissure diathermy (*n* = 53; 31.4%), condyloma excision (*n* = 17; 10.1%), and fistulotomy/fistulectomy (*n* = 5; 3.0%). The mean in-hospital stay was 1.6 days (SD 1; range, 1–4); it was prolonged beyond one day in 20 patients (4.6%) due to mild bleeding or postoperative pain, representing the more frequent early postoperative complications (Table 2).

After stratification by the extent of the internal rectal prolapse, the mean volume of the doughnuts was significantly higher (13.8 mL; SD, 1.5) in the group of 341 patients with an internal rectal prolapse exceeding more than half of the length of the CAD than in the group of 89 patients with smaller prolapse (8.9 mL; SD, 0.7) (*P* value <0.05) (Table 3).

As regards follow-up data at six months, residual haemorrhoidal disease occurred in eight out of 430 patients (1.8%), with six of them (75%) having originally a large internal rectal prolapse. Moreover, a high index of patient satisfaction (VAS = 8.3; SD, 1.2) and a clinically relevant reduction of the constipations scores (CSS = 6.0; SD, 2.6) were reported (Table 4). Recurrent haemorrhoidal disease was detected at 12-month follow-up in five out of 254 patients (1.9%), with all of them having originally a large internal rectal prolapse; again, a high index of patient satisfaction (VAS = 8.9; SD, 0.9) coupled with a persistent reduction of constipation scores (CSS = 5.0; SD, 2.2) was observed (Table 5).

## 4. Discussion

SH represents an innovative surgical treatment of haemorrhoids not only for the technical details of the operation, which avoids any wound in a very sensitive area such as the anus and perianal skin, but also for its new pathophysiological concept aimed at the correction of the internal rectal prolapse thought to determine the sliding down of the haemorrhoids from the anal canal. Actually, this operation simply “lifts” the haemorrhoids back into their original anatomic site by means of the circular excision of a variable volume of rectal wall (mucosa-submucosa with contiguous muscular fibres); this is accomplished with a circular stapler that allows both the transverse transection of rectal tissue and the end-to-end anastomosis at least 2-3 cm from the dentate line [17].

The advantages of SH over CH are confirmed by sound clinical data with less postoperative pain, superior functional recovery, and earlier return to normal activities, improved patient satisfaction coupled with a significant improvement of obstructed defecation symptoms. Unlikely, the risk of residual/recurrent haemorrhoids is two- to threefold higher after SH as compared to CH, with a rate up to 29.4% in patients with large internal rectal prolapse [10, 12, 18].

For these reasons, STARR has been proposed as an alternative to SH in patients with large internal rectal prolapse, that is, a prolapse exceeding half or more of the longitudinal length of the CAD at the intraoperative assessment. Clinical studies have confirmed the significant reduction of residual/recurrent disease by means of the STARR procedure; noteworthy, the recurrence rate in the specific subset of patients with a large internal rectal prolapse was reduced to 1.9–5.9% [12, 13, 19]. This improvement seems to be mainly related to the wider extent of prolapse resection amenable with the STARR procedure. Conversely, neither the details of the operation* per se* nor the deeper rectal wall resection accomplished with STARR may justify these results, also because the historical distinction between SH and STARR based on the thickness of the doughnuts (mucosal versus full-thickness rectal wall resection) has lost much of its clinical relevance as smooth muscle fibres can be found in 4% to 97% of excised mucosal rings after SH [20].

So, there was primarily the need of new HV staplers, such as the CPH34 HV, in order to guarantee higher volumes of prolapse resection as compared to previously available devices so as to avoid a double rectal resection. Actually, CPH34 HV has an enlarged stapler housing volume with a cylindrical shape that was obtained by lowering the internal tissue stop and by thinning and strengthening the anvil shaft of the stapler. These technological improvements, coupled with smoothed angles at the edge of the tissue storing area, allow for a better introduction of the prolapsed tissue within the stapler casing that translates into a more efficient use of the whole theoretically available stapler casing volume. Moreover, the haemostatic properties of the stitches were improved by using 32 circumferentially mounted staples, thus achieving a better control of bleeding at the suture line.

Following experimental testing that confirmed both higher volumes of resection and less anastomotic bleeding, a retrospective multicentric clinical study was performed in order to verify the safety and the efficacy of CPH34 HV, especially in terms of postoperative bleeding and extent of prolapse resection [14]. As regards bleeding complications, a minority of patients required only very few haemostatic stitches (1.3/patient: range = 0–7) for the intraoperative control of anastomotic bleeding: mostly, a minor postoperative bleeding that was well managed with conservative measures occurred in 12 patients (2.8%) only, while this is usually reported in almost 30% of patients, thus confirming the more than satisfactory haemostatic properties of CPH34 HV [8].

Moreover, our follow-up data confirmed the effectiveness of CPH34 HV as regards the adequacy of prolapse resection as suggested not only by the high mean volume of the doughnuts but, most of all, by the low rate of residual disease. Actually, in a study population including 79.3% of patients bearing a large internal rectal prolapse only eight out of 430 patients (1.8%) had persistence of haemorrhoidal prolapse within six months after the operation, even better than most of the previous clinical experiences with the STARR procedure [12, 13, 19]. Similarly, the rate of haemorrhoidal recurrence was very low (*n* = 5, 1.9%); the observation that each patient with haemorrhoidal recurrence had originally a large rectal prolapse would confirm the key role of the extent of rectal prolapse as one of the most relevant risk factors for haemorrhoidal recurrence, thus emphasizing the need to perform a more than complete prolapse resection. Overall, residual/recurrence haemorrhoids were reported in less than 4% of patients, which means at least a five- to sixfold reduction of postoperative haemorrhoidal prolapse as compared to previous clinical experiences, especially in patients with a large internal rectal prolapse (25–29.4%) [10, 12, 13]. Our favourable clinical findings are well explained by the high mean volume of the doughnuts (12.7 mL) that was almost double as compared to those retrieved after SH was performed with conventional devices (6-7 mL) [16]. Worth noting, specimen volumes up to 18 mL were observed in patients with a large rectal prolapse, thus meaning that approximately 72% of the maximum theoretical volume (25 cm^3^) of the stapler casing of CPH34 HV is “really” available for prolapse resection while with the traditional PPH03-33 (17.4 cm^3^) no more than 40% of the casing can be available for prolapse resection [13].

Finally, as regards another frequent early postoperative complaint in patients undergoing SH, that is spontaneous and/or postdefecation anal pain, this symptom was seldom reported (6.5%) in our experience and, when occurring, it was usually well controlled with mild analgesics with no need of hospital readmission, which is reported approximately in 1.7% of patients [7, 21]. This finding, coupled with the low rate of faecal urgency (4.7%) as compared to previous experiences (13.7%–25.1%), seems to be related to a frequently neglected detail of the operation, namely, the “controlled digital stretching” prior to the insertion of the operating proctoscope [18, 22]. As a matter of fact, this preliminary manoeuvre aids in reducing the high preoperative anal pressure which is frequently reported in patients with haemorrhoidal disease and that may impair the ability to satisfactorily evacuate the rectum in the early postoperative period [23].

## 5. Conclusions

The interim analysis of this retrospective multicentric clinical study in patients undergoing SH by means of CPH34 HV for haemorrhoids, with a high prevalence of associated large internal rectal prolapse, suggests that the higher volume of the doughnuts well correlated with a clear-cut reduction of both residual and recurrent haemorrhoidal prolapse, which translated into a high index of patient satisfaction and clinically relevant reduction of constipations scores. Moreover, both intra- and early postoperative bleeding complications were seldom reported, thanks to the haemostatic properties of this new stapler device; complete follow-up data will define further the safety and efficacy of this new stapler device.

## Figures and Tables

**Table 1:** Clinical characteristics of patients (*N* = 430).

		*N*	%
Age, yrs			
Mean (SD)	51 (13.4)		
Range	19–80		
Sex			
Male		209	48.6
Female, *n* (%)		221	51.4
Specific symptoms:			
Pain score (VAS: 0–10)			
Mean (SD)	4.2 (2.1)		
Range	0–10		
Bleeding, *n* (%)		363	84.4
Haemorrhoidal prolapse, *n* (%)		363	84.4
Constipation, *n* (%)		190	44.2
Soiling, *n* (%)		57	13.3
Diarrhoea, *n* (%)		53	12.3
Goligher's Classification:			
III		169	39.3
IV		261	60.5
Constipation Scoring System			
Mean (SD)	9.3 (3.6)		
Range	1–15		
Previous anorectal surgery		55	12.8

SD: Standard Deviation.

**Table 2:** Intra- and early postoperative findings (*N* = 430 patients).

		*N*	%
Operative time, minutes			
Mean (SD)	26.1 (6.9)		
Range	15–60		
Prolapse involving more than half of the length CAD			
No		89	20.7
Yes		341	79.3
Type of Prolapsectomy			
Traditional “Stapled Anopexy”		394	91.6
“Parachute” Technique		36	8.4
Haemostatic stitches, *n*			
Mean (SD)	1.3 (1.7)		
Range	0–7		
Technical failures of the device		1	0.2
Associate procedures, *n* (%)		168	39.0
Skin Tags Excision		73	43.4
Anal Fissure		53	31.4
Condiloma		17	10.2
Fistulotomy/Fistulectomy		5	3.0
Miscellaneous		20	12.0
Hospital stay, days			
Mean (SD)	1.6 (1.0)		
Range	1–4		
Early post-operative complications		62	14.4
Anal pain (spontaneous/Postdefecation)		28	6.5
Bleeding		12	2.8
Acute urinary retention		5	1.2
Urgency		14	3.3
Thrombosed haemorrhoids		1	0.2
Others		2	0.4
Re-operation (within 30 days)		2	0.4

SD: Standard Deviation.

**Table 3:** Specimen measures stratified by type of prolapsectomy (traditional Longo’s procedure or “Parachute” Technique) and extent of rectal prolapsed.

	Mean (SD)	Range
Total patients (n=430)		
Length, mm	82.8 (11.3)	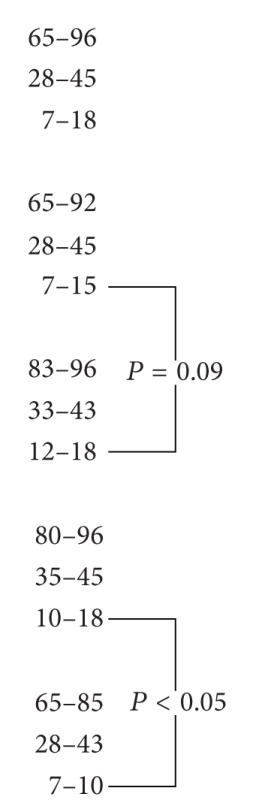
Height, mm	37.5 (4.3)	
Volume, mL	12.7 (2.1)	
Stapled Anopexy (n=394)		
Length, mm	82.5 (11.3)	
Height, mm	37.4 (4.3)	
Volume, mL	12.4 (2.1)	
“Parachute” Technique (n=36)		
Length, mm	85.6 (5.2)	
Height, mm	38.7 (2.3)	
Volume, mL	15.1 (1.8)	
Prolapse more than half of CAD (n=341)		
Length, mm	85.0 (5.6)	
Height, mm	38.4 (3.9)	
Volume, mL	13.8 (1.5)	
Prolapse less than half of CAD (n=89)		
Length, mm	75.0 (16.3)	
Height, mm	34.7 (4.3)	
Volume, mL	8.9 (0.7)	

SD: Standard Deviation.

**Table 4:** Follow-up at six months in 430 patients.

		*N*	%
*Residual disease (within six months) *			
Spontaneous pain score (VAS: 0–10)			
Mean (SD)	1.6 (1.2)		
Range	1–7		
Pain at defecation (VAS: 0–10)			
Mean (SD)	1.8 (1.2)		
Range	1–7		
Bleeding, *n* (%)		6	1.4
Residual haemorrhoidal prolapse		8	1.8
Other symptoms/signs			
Urgency		20	4.7
Pruritus		2	0.4
Soiling		1	0.2
Incontinence		0	—
Anal stenosis		1	0.2
Anal fissure/abscess/fistula		0	—
Haemorrhoidal thrombosis		1	0.2
Residual skin tags		10	2.3
Patient satisfaction (VAS: 0–10)			
Mean (SD)	8.3 (1.2)		
Range	2–10		
Constipation Scoring System			
Mean (SD)	6.0 (2.6)		
Range	0–14		

SD: Standard Deviation.

**Table 5:** Follow-up at 12 months in 254 patients.

		*N*	%
*Recurrent disease (after six months) *			
Spontaneous pain score (VAS: 0–10)			
Mean (SD)	1.3 (0.8)		
Range	1–3		
Pain at defecation (VAS: 0–10)			
Mean (SD)	1.2 (0.4)		
Range	1-2		
Bleeding, *n* (%)		1	0.4
Residual haemorrhoidal prolapse		5	1.9
Other symptoms/signs			
Urgency		3	1.2
Pruritus		1	0.4
Soiling		1	0.4
Incontinence		0	—
Anal stenosis		0	—
Anal fissure/abscess/fistula		0	—
Haemorrhoidal thrombosis		1	0.4
Residual skin tags		12	4.7
Patient satisfaction (VAS: 0–10)			
Mean (SD)	8.9 (0.9)		
Range	6–10		
Constipation Scoring System			
Mean (SD)	5.0 (2.2)		
Range	0–13		

SD: Standard Deviation.

## References

[1] Loder PB, Kamm MA, Nicholls RJ, Phillips RKS (1994). Haemorrhoids: pathology, pathophysiology and aetiology. *The British Journal of Surgery*.

[2] Landolfi V, Stuto A, Carriero A, Longo A (2007). Emorroidi e prolasso. *Ospedali D'italia Chirurgia*.

[3] Longo A Treatment of haemorrhoids disease by reduction of mucosa and haemorrhoidal prolapse with a circular suturing device: a new procedure.

[4] Mehigan BJ, Monson JRT, Hartley JE (2000). Stapling procedure for haemorrhoids versus Milligan-Morgan haemorrhoidectomy: randomised controlled trial. *The Lancet*.

[5] Shalaby R, Desoky A (2001). Randomized clinical trial of stapled versus Milligan-Morgan haemorrhoidectomy. *The British Journal of Surgery*.

[6] Boccasanta P, Capretti PG, Venturi M (2001). Randomised controlled trial between stapled circumferential mucosectomy and conventional circular hemorrhoidectomy in advanced hemorrhoids with external mucosal prolapse. *The American Journal of Surgery*.

[7] Ng KH, Ho KS, Ooi BS, Tang CL, Eu KW (2006). Experience of 3711 stapled haemorrhoidectomy operations. *The British Journal of Surgery*.

[8] Sutherland LM, Burchard AK, Matsuda K (2002). A systematic review of stapled hemorrhoidectomy. *Archives of Surgery*.

[9] Slawik S, Kenefick N, Greenslade GL, Dixon AR (2007). A prospective evaluation of stapled haemorrhoidopexy/rectal mucosectomy in the management of 3rd and 4th degree haemorrhoids. *Colorectal Disease*.

[10] Tjandra JJ, Chan MKY (2007). Systematic review on the procedure for prolapse and hemorrhoids (stapled hemorrhoidopexy). *Diseases of the Colon and Rectum*.

[11] Reboa G, Gipponi M, Logorio M, Marino P, Lantieri F (2009). The impact of stapled transanal rectal resection on anorectal function in patients with obstructed defecation syndrome. *Diseases of the Colon and Rectum*.

[12] Boccasanta P, Venturi M, Roviaro G (2007). Stapled transanal rectal resection versus stapled anopexy in the cure of hemorrhoids associated with rectal prolapse. A randomized controlled trial. *International Journal of Colorectal Disease*.

[13] Naldini G, Martellucci J, Talento P, Caviglia A, Moraldi L, Rossi M (2009). New approach to large haemorrhoidal prolapse: double stapled haemorrhoidopexy. *International Journal of Colorectal Disease*.

[14] Reboa G, Gipponi M, Testa T, Lantieri F (2011). Technological improvements in the treatment of haemorrhoids and obstructed defaecation syndrome. *In Vivo*.

[15] Agachan F, Chen T, Pfeifer J, Reissman P, Wexner SD (1996). A constipation scoring system to simplify evaluation and management of constipated patients. *Diseases of the Colon and Rectum*.

[16] Caviglia A, Mongardini M, Malerba M (2011). Single Stapler Parachute Technique (SSPT): a new procedure for large hemorroidal prolapse. *Il Giornale di Chirurgia*.

[17] Corman ML, Gravié JF, Hager T (2003). Stapled haemorrhoidopexy: a consensus position paper by an international working party—indications, contra-indications and technique. *Colorectal Disease*.

[18] Shao WJ, Li GCH, Zhang ZHK, Yang BL, Sun GD, Chen YQ (2008). Systematic review and meta-analysis of randomized controlled trials comparing stapled haemorrhoidopexy with conventional haemorrhoidectomy. *The British Journal of Surgery*.

[19] Stuto A, Favero A, Cerullo G, Braini A, Narisetty P, Tosolini G (2012). Double stapled haemorrhoidopexy for haemorrhoidal prolapse: indications, feasibility and safety. *Colorectal Disease*.

[20] Naldini G, Martellucci J, Moraldi L, Romano N, Rossi M (2009). Is simple mucosal resection really possible? Considerations about histological findings after stapled hemorrhoidopexy. *International Journal of Colorectal Disease*.

[21] Butterworth JW, Peravali R, Anwar R, Ali K, Bekdash B (2012). A four-year retrospective study and review of selection criteria and post-operative complications of stapled haemorrhoidopexy. *Techniques in Coloproctology*.

[22] Carriero A, Longo A (2003). Complicanze intraoperatorie, perioperatorie e post-operatorie della emorroidopessi con suturatrice meccanica. *Ospedali D'italia Chirurgia*.

[23] Arabi Y, Alexander Williams J, Keighley MRB (1977). Anal pressures in hemorrhoids and anal fissure. *The American Journal of Surgery*.

